# AZO/PEDOT:PSS Polymer Frontal Interface Deposited on Flexible Substrates for a-Si:H Photovoltaic Applications

**DOI:** 10.3390/polym10101068

**Published:** 2018-09-27

**Authors:** Svetlana Mansurova, Ismael Cosme, Andrey Kosarev, Antonio J. Olivares, Carlos Ospina, Hiram E. Martinez

**Affiliations:** 1 National Institute for Astrophysics, Optics, and Electronics (INAOE), Puebla 72840, Mexico; smansur@inaoep.mx (S.M.); akosarev@inaoep.mx (A.K.); adj.olivares@gmail.com (A.J.O.); c_ao_o@yahoo.es (C.O.); hiram@inaoep.mx (H.E.M.); 2 CONACyT–INAOE, Puebla 72840, Mexico

**Keywords:** PEDOT:PSS polymer, flexible substrate, photovoltaic, hybrid inorganic-polymer

## Abstract

Thin-film hybrid organic-inorganic photovoltaic structures based on hydrogenated silicon (Si:H), poly(3,4ethylenedioxythiophene):poly(4-styrenesulfonate) (PEDOT:PSS) polymer Al-doped ZnO (AZO) films deposited on different types of flexible substrates have been fabricated and investigated. The compatibility of the polymer and inorganic materials regimes and deposition techniques used for device fabrication has been demonstrated on flexible substrates. Morphological characteristics of transparent Al-doped ZnO (AZO) films deposited on substrates have been measured by atomic force microscopy. Electronic characteristics of the fabricated photovoltaic structures have been measured and analyzed for different thicknesses of the transparent electrodes and different substrate types. Photovoltaic hybrid structure on polyethylene naphthalate (PEN) substrate showed the best characteristics: short circuit current density *J*_sc_ = 9.79 mA/cm^2^, open circuit voltage *U*_oc_ = 565 mV, and PCE η = 1.3%. To analyze the mechanisms governing the device performance, short circuit current density spectral dependence of the devices fabricated on different types of flexible substrates has been measured. As demonstrated by our analysis, the structures on PEN substrates, besides better substrate transmittance, also show better junction properties.

## 1. Introduction

Photovoltaic (PV) structures on lightweight and flexible substrates have several advantages over the heavy glass-based structures in both terrestrial and space applications [1,2]. The cells mounted on flexible foil are not fragile, the requirements of the supporting structures are minimal, and they can be wrapped onto any suitably oriented or curved structures. The specific power of the solar cells is an important factor in space applications and hence the development of photovoltaic devices on light-weight substrates is interesting.

Third generation organic photovoltaics, which include devices with flat and bulk heterojunction between the various types of conjugated polymers, small molecules, fullerene derivatives, and carbon nanotubes, continue to attract great attention from the scientific community [3,4,5]. Solution-processed organic solar cells offer the attractive feature of providing low-cost, high-efficiency, flexible, and large-area photovoltaic devices through cheap roll-to-roll fabrication at a low processing temperature [6]. As a result of impressive efforts in developing novel low bandgap polymer donor materials, optimizing the photoactive layer morphology, interface engineering, and novel light-trapping structures [7,8,9,10,11,12], single-junction organic solar cells are now approaching the practical efficiency limit of η ≈ 11%–12% [13]. Improving the efficiency of polymer solar cells beyond 15% is required to be able to compete with other thin-film photovoltaic technologies.

An alternative to pure organic PV cells is the class of organic-inorganic hybrid solar cells [14,15,16,17], where heterojunction is formed between inorganic semiconductors and organic compounds. An advantage of hybrid over organic PV devices lies in the high carrier mobility of the inorganic semiconductor and the higher light absorption in the longer wavelength range than that of organic compounds. In addition, bandgap engineering can be a useful instrument in the design of the hybrid solar cell architecture since rather developed deposition process of inorganic semiconductors allows tuning the structural and electronic parameters such as optical gap, Fermi level position, localized states distribution, etc.

Hydrogenated silicon (Si:H) prepared by plasma enhanced chemical vapor deposition (PECVD) method is a widely known inorganic semiconductor with a rather mature deposition technology which has been successfully implemented in several commercial electronic and optoelectronic devices, including photovoltaics [18]. This material is also used for large area applications; it can be deposited on top of an arbitrarily shaped surface, and, therefore, becomes a potential candidate for flexible solar cells.

Even though organic-inorganic hybrid solar cells on glass substrates have shown the efficiencies exceeding 12%, there are no reports on the development of these devices on flexible substrates. Here we present the results of an investigation of thin-film hybrid organic-inorganic photovoltaic structures based on flat heterojunction hydrogenated silicon (Si:H) and poly(3,4 ethylene dioxythiophene):poly(4-styrenesulfonate) (PEDOT:PSS) fabricated on different types of flexible substrates, namely, polyethylene naphthalate (PEN) and poly (4,4′-oxydiphenylene-pyromellitimide) (“Kapton”).

Performance characteristics of fabricated photovoltaic structures have been measured and analyzed for different thicknesses of the transparent electrodes and different substrate types. The goal of this work is (i) to reveal problems of fabrication (compatibility of PECVD-based films, polymer semiconductor and transparent conductive oxides deposited by sputtering with each other and with plastic substrate, etc.); (ii) to study the fabricated device structures; and (iii) to analyze the impact of the frontal interface morphological properties on the device performance.

## 2. Materials and Methods

The choice of an appropriate substrate is a crucial factor for hybrid flexible solar cells in the superstrate configuration (illumination through the substrate) because the substrate should be optically transparent and should withstand the processing temperature (in the range of 150–200 °C) and the glow discharge conditions during deposition of semiconductor films in PECVD system. For this purpose, two types of flexible substrates—PEN (Tejin Inc., Osaka, Japan), and “Kapton” (DuPont, Inc., Wilmington, DE, USA)-have been selected. PEN-based films are well-known substrates widely used in a range of electronic applications and they possess a number of advantages, such as excellent dimensional stability, low moisture pick up, good solvent resistance, and high optical transparency. “Kapton”, in its turn, is characterized by high mechanical and thermal stability and, as such, it can withstand glow discharge and high temperatures (~450 °C) and might be suitable for space applications [1]. However, one of the main disadvantages of “Kapton” is a strong absorption of visible radiation

**Figure 1:**
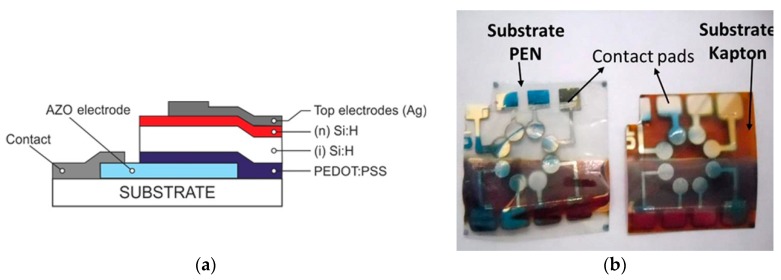
(**a**) Hybrid photovoltaic structure on a flexible substrate based on hydrogenated silicon (Si:H) and organic semiconductors poly(3,4 ethylene dioxythiophene):poly(4-styrenesulfonate) (PEDOT:PSS) as p-type layer; (**b**) photo of the fabricated structures on both polyethylene naphthalate (PEN) and Kapton substrates.

The sequence of deposited layers follows the structure presented schematically in Figure 1a. The device configuration studied is similar to a-Si:H based p–i–n structure where p-layer is substituted by organic semiconductor PEDOT:PSS.

As the transparent electrode the Al:ZnO (AZO) films were deposited on top of a flexible substrate by radio frequency (RF) magnetron sputtering from ceramic (15 × 5 cm^2^) ZnO target with 2 wt % of Al_2_O_3_ using the sputtering module of 4-chamber equipment from MVSystem Inc. (Golden, CO, USA). This installation is a semi-industrial commercial system allowing fabrication of large area (15 × 15 cm^2^) multilayered optoelectronic structures based on different materials deposited by plasma (in three chambers) or by sputtering (one chamber with up to three targets) with uniformity of 94%. The deposition was performed in RF discharge in argon with power loaded W = 100 W and pressure P = 2 mTorr. Thin films (255–510 nm thick) were deposited at substrate temperature *T*_s_ = 175 °C, suitable for a plastic substrate. The AZO films were etched by hydrochloric acid to define the bottom electrode and cleaned with ultrasound baths in acetone and isopropyl alcohol, sequentially. The PEDOT:PSS blend mixtures were prepared from components purchased from Ossila Ltd. (Sheffield, UK). Prior to deposition, the PEDOT:PSS mixture (1:6 weight ratio) was filtered with a PVDF (polyvinylideneflouride) filter with pore sizes of 0.45 µm. PEDOT:PSS films with a thickness of 250 nm were deposited by spin-coating at a rotation speed of 2000 rpm in N_2_ ambient.

The inorganic layers were deposited using a cluster multi chamber PECVD system from MVSystem Inc. with RF discharge at frequency f = 13.56 MHz. To avoid cross contamination the doped and intrinsic layers were deposited in different chambers with transportation via vacuum camera. The intrinsic Si:H layers were deposited from a 10% SiH_4_ + 90% H_2_ gas mixture at pressure P = 550 mTorr with deposition time selected to obtain required thickness. The 20 nm thick n-layers were deposited using 0.01% PH_3_ + 9.9% SiH_4_ + 90.09% H_2_ gas mixtures at pressure P = 550 mTorr. The deposition temperature was fixed at *T*_d_ = 160 °C and power at W = 3 Watt. All the gases used for deposited semiconductor films were semiconductor grade from Matheson Inc. (Sacramento, CA, USA).

The top contacts were deposited by evaporation or sputtering of Ag through a metal shadow mask with an area of *S*_m_ = 0.09 cm^2^. Photos of the resulting hybrid structures on PEN and “Kapton” flexible substrates are shown in Figure 1b.

For each substrate type, two devices AZO/PEDOT:PSS/(i)Si:H-(n)Si:H with different AZO electrode thickness have been fabricated: PEN/AZO(255 nm)/PEDOT:PSS/(i)Si:H-(n)Si:H labeled as H42-2; PEN/AZO(510 nm)/PEDOT:PSS/(i)Si:H-(n)Si:H labeled as H43-2; Kapton/AZO(255 nm)/PEDOT:PSS/(i)Si:H-(n)Si:H labeled as H44-2; Kapton/AZO(510 nm)/PEDOT:PSS/(i)Si:H-(n)Si:H labeled as H45-2. In addition, for comparison purposes, a reference AZO/PEDOT:PSS/(i)Si:H-(n)Si:H device on “Corning 1737” glass (labeled as H41-1) has been fabricated. We have selected 255 nm and 510 nm AZO thicknesses for analysis due to 255 nm was the optimal thickness used for structures based on solar cell on glass substrates and 510 nm thickness has resulted to be the functional thickness for a flexible substrate. Below 510 nm thickness, the results were ambiguous for application on the solar cell device due to the variation of defects in flexible substrates.

The current density–voltage characteristics were measured by using a “Keithley 6517A” electrometer. The samples were illuminated under standard AM1.5 conditions provided by a solar simulator “Oriel 2A” from Newport Corp. (Irvine, CA, USA) and the current-voltage J(U) curves were analyzed for different samples to extract the solar cells characteristics. Spectral dependence of short circuit current density was measured using a xenon and halogen lamp illumination with a “Triax320” monochromator calibrated with a thermopile sensor mod.71938 from Thermo-Oriel Instruments Inc. (London, UK) and current response by “Keithley 6517A” electrometer. During the spectral measurements, the samples were illuminated with monochromatic light of *I*_0_ = 4 mW/cm^2^ intensity in the wavelength range from λ = 350 to 1200 nm with a wavelength resolution Δλ = 3.96 nm. Morphological characteristics of substrates and AZO layers have been investigated using an Easy scan atomic force microscope (AFM, Easy Scan 2, Nano surf Inc., Liestal, Switzerland) in semi-contacting mode. Scanning electron microphotography of the device structure was obtained in a secondary electron regime using “FEI Scios” Dual Beam scanning electron microscope with a focused ion beam for preparation of the samples.

## 3. Results

### 3.1. Optical Characterization of Substrates

**Figure 2:**
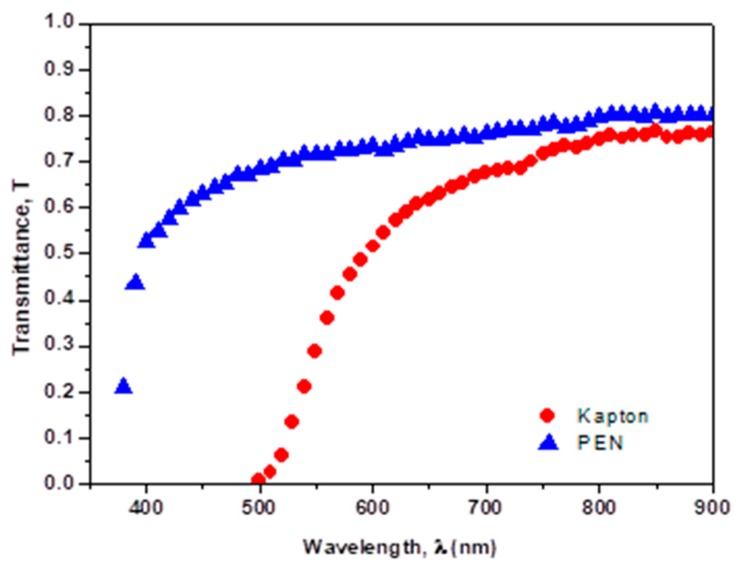
Absorption spectra of the flexible substrates (PEN and “Kapton”) used for fabrication of photovoltaic structures.

Optical transmission spectra *T*(λ) of the PEN and “Kapton” films used in this study are shown in Figure 2.

The optical transmission of the PEN substrate is quite flat and it is around 70% within the visible range (λ ≈ 400–900 nm). Compared with PEN, “Kapton” substrate is opaque for the short wavelength region of spectra (below λ = 500 nm), then its transmittance gradually increases to 65% in the range of λ = 500 to 700 nm and remains constant at this value in the wavelength range λ ≈ 700–900 nm.

### 3.2. Surface Morphology

Unlike glass, polymer substrates cannot be polished and the surface morphology can affect significantly the electronic properties and photovoltaic characteristics [1]. The reason for this relies on the fact that the process of film growth, and, as a consequence, the film morphology in thin-film-based photovoltaic devices is sensitive to the morphology of the surface of the preceding layer where the film growth starts, affecting in this way both, an individual film properties as well as junction properties.

**Figure 3:**
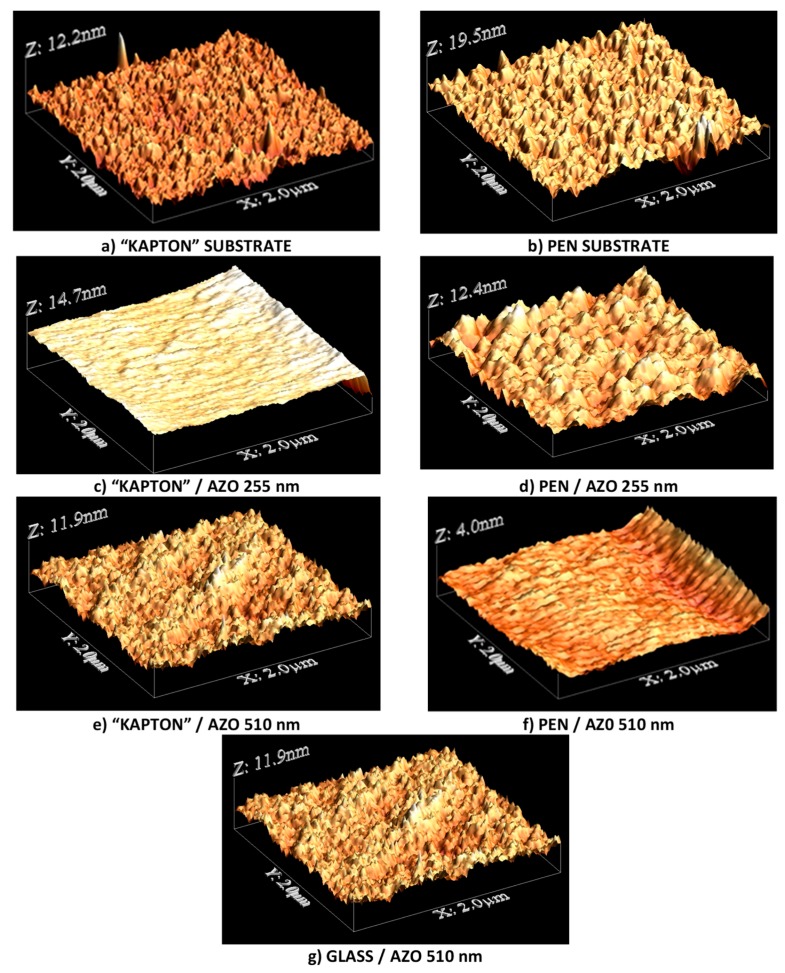
Atomic force microscopy images of (**a**) “Kapton” substrate; (**b**) PEN substrate; (**c**) 255 nm-thick Al:ZnO (AZO) film on “Kapton”; (**d**) 255 nm-thick AZO film on PEN; (**e**) 510 nm-thick AZO film on “Kapton”; (**f**) 510 nm-thick AZO film on PEN and (**g**) 510 nm-thick AZO film on a glass substrate.

Surface morphological characteristics were measured by atomic force microscopy imaging of the AZO thin films grown on PEN and “Kapton” substrates and compared with reference data for the substrates without AZO. The AFM images (Figure 3) were statistically processed to obtain surface morphological characteristics, such as root mean square roughness *R*_q_ (statistical momentum of the second order of height distribution), skewness *S*_k_ (third order momentum), and kurtosis *K*_u_ (fourth order momentum). Skewness tells us whether the distribution function is symmetric or skewed, and it reveals whether the surface morphology is hill dominated (*S*_k_ > 0) or valley dominated (*S*_k_ < 0). *K*_u_, in its turn, describes the sharpness of the distribution function and it reveals whether the morphology of the surface is dominated by sharp or bumpy shapes. For *K*_u_ < 3 the central peak of the distribution function is broader and lower than normal with relative shorter tails (more uniformed distribution) that is related to a surface dominated by bumpy hills (or valleys, depending on *S*_k_). For *K*_u_ > 3 the central peak of the distribution function is higher and sharper than normal (Gauss) and the tails are longer meaning a surface morphology dominated by sharp peaks/pores.

The results of the evaluation of statistical parameters *R*_q_, *S*_k_, and *K*_u_ are presented in Table 1. Some differences can be observed in characteristics of the substrate surfaces: PEN, as compared to “Kapton”, is characterized by slightly higher *R*_q_; its *S*_k_ is positive, meaning hill dominated surface, while “Kapton” value is negative, meaning valley dominated surface, though both values of *S*_k_ are rather small (<0.5) which statistically means an approximately symmetrical distribution [19]. The values of *K*_u_ for PEN and “Kapton” surfaces are slightly smaller and larger than 3 (2.84 and 3.42 correspondingly), which reveals that morphological features of “Kapton” are somewhat sharper than those of PEN.

Deposition of 255 nm AZO layer on top of different substrates results in different AZO surface morphologies. For a PEN-based AZO surface a small increase of roughness *R*_q_ is observed (14%), hills keep dominating (still slightly) and they become sharper than before (*K*_u_ = 3.15). The “Kapton”-based AZO surface also becomes rougher (72%); however, the morphology changes to that of hill dominated (*S*_k_ changes its sign) and the surface morphology exhibits increased sharpness (*K*_u_ = 4.8).

Further evolution of the morphology of AZO deposited on different substrates at the increased AZO thickness (to 510 nm) can be described as follows. Both, PEN- and “Kapton”-based AZO surfaces display a remarkable decrease in roughness (29% and 24% correspondingly). For PEN-based AZO surface, now valleys become slightly dominating and low value of the kurtosis (*K*_u_ < 3) indicates that they are bumpier now. For “Kapton”-based AZO surface the hills now definitely prevail (rather large value of *S*_k_ > 1 points out highly skewed distribution [19]), and a remarkably large value of the kurtosis (*K*_u_ = 7.6) indicates that the surface morphology shows significantly increased sharpness (spike-like features). For comparison, a 510 nm-thick AZO film has been deposited on a glass substrate (Figure 3g). It is observed that similar values of RMS were reached for the films deposited on a glass substrate and flexible substrates. In the case of *S*_k_ and *K*_u_, the AZO film deposited on polyimide show more deviation from that obtained on a glass substrate (Table 1).

It is worth noting that the evolution of surface morphology is rather poorly reported in the literature. We think that characteristics of surface morphology influence device properties, comprising many layers fabricated by means of different techniques. Although the problem is of much importance, in this work, we are limited only by AFM data and SEM image for illustration.

### 3.3. SEM Imaging

**Figure 4:**
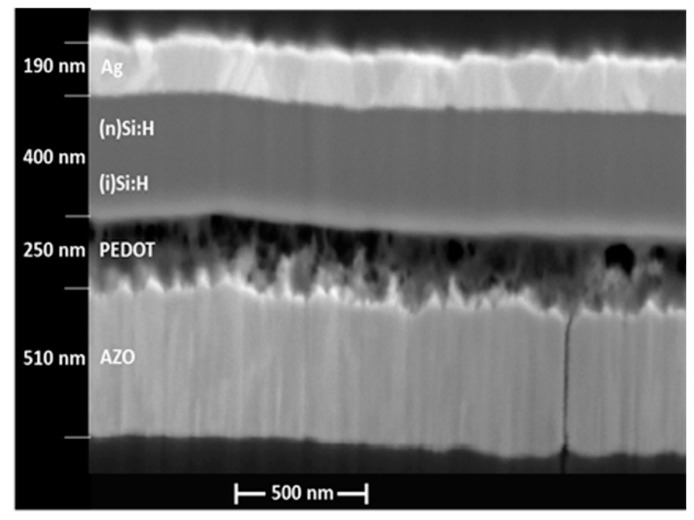
Cross-sectional scanning electron microscopy (SEM) image of a representative device.

The cross-section scanning electron microscopy (SEM) image (from secondary electron regime) of the hybrid photovoltaic structure H44 is presented in Figure 4. It is seen, that the AZO layer has a well-defined columnar structure with a column width of approximately W_col_ = 10–50 nm. PEDOT:PSS layer, deposited on AZO is rather inhomogeneous showing spherical inclusions, mainly at the PEDOT:PSS/a-Si boundary. It can be seen, however, that PEDOT layer “heals” rather rough top AZO surface, preparing a smooth and planar surface for the deposition of an intrinsic amorphous silicon layer. Thus, a-Si layer is deposited on a better surface and looks rather homogeneous in thickness and structure (at the given resolution). Next, the Ag layer, demonstrates a well-defined grain structure (with inverted pyramid form) and the grain size of around 50–150 nm. It is interesting to note that the substrate defect (crack) is translated through the AZO layer (either during the deposition or due to the substrate bending) and it is interrupted due to the organic polymer layer which prevents the further translation of the defect to the subsequent absorbing layers.

### 3.4. Current-Voltage Characteristics

**Figure 5:**
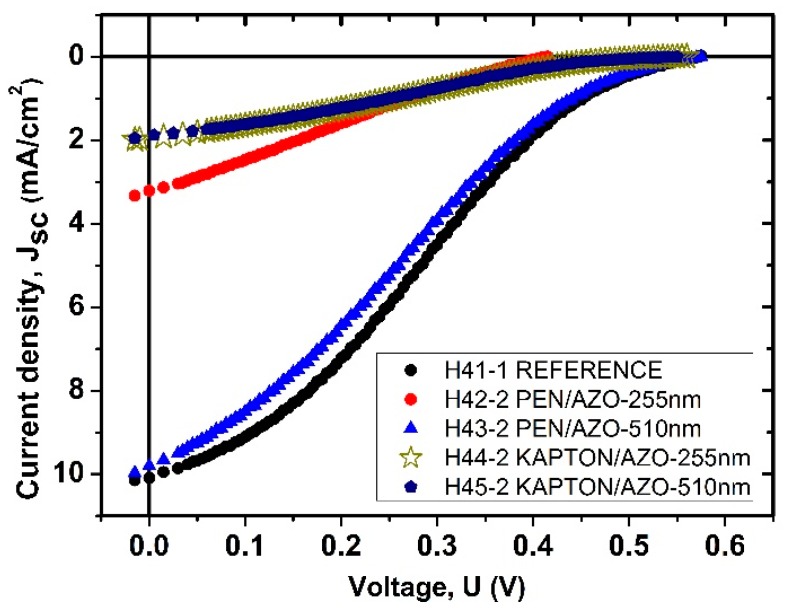
Current-voltage characteristics of hybrid photovoltaic structures: reference sample on “Corning 1737” glass (H41-1), AZO/PEDOT:PSS/(i)Si:H-(n)Si:H deposited on different substrates with different thickness of AZO electrodes: PEN/AZO(255 nm)/PEDOT:PSS/(i)Si:H-(n)Si:H (H42-2), :PEN/AZO(510 nm)/PEDOT:PSS/(i)Si:H-(n)Si:H (H43-2), Kapton/AZO(255 nm)/PEDOT:PSS/(i)Si:H-(n)Si:H (H44-2), Kapton/AZO(510 nm)/PEDOT:PSS/(i)Si:H-(n)Si:H (H45-2).

In order to study the electronic properties of our samples, the J(U) characteristics of the AZO/(p)PEDOT:PSS/(i)Si:H/(n)Si:H structures fabricated on PEN and “Kapton” substrates were measured (Figure 5). For comparison purpose, the *J*(*U*) characteristics of the cell prepared on a glass substrates in the same technological run were measured in similar experimental conditions. For both substrates, the *J*–*U* curves of the devices measured under standard illumination (AM 1.5 *I*_0_ = 100 mW/cm^−2^) from solar simulation are shown in Figure 5 and the corresponding photovoltaic performance characteristics are summarized in Table 2.

Let us start with the analysis of the *J*(*U*) curves in Figure 5 with *J*_sc_. The highest values are observed in the samples deposited on PEN substrates with minor difference between the two samples due to the different thickness of the AZO layer. *J*_sc_ values in the samples fabricated on “Kapton” substrates are significantly less which is expected due to the poor transmission T(λ) in the short wavelength region. Besides, the electronic properties of junctions fabricated on different substrates with different surface morphology (see AFM data in Table 2) could also be responsible for this differences (will be discussed later). As to the *U*_oc_ values all the structures have close values *U*_oc_ = 0.55–0.565 V, with the exception of sample H42-2 with *U*_oc_ = 0.405 V.

It can be seen that the *J*_sc_ of the fabricated devices shows a limitation of current in the range of *U* = 0.3–*U*_oc_ (an S-shape of the *J*_sc_ (*U*) curve near *U* = *U*_oc_). Therefore, low fill factor (FF) values (from 23% to 26%) are observed in all the structures resulting from losses of current in these not optimized structures. Low FF often arises from parasitic shunt and series resistances and might be a signal of leakage through the organic layer or/and other semiconductor layers (see SEM image in Figure 4 for possible current channels). However, at present it is not clear yet if and how plasma could have an influence on inducing such current channels.

It is observed, that performance characteristics of devices fabricated on PEN substrate are dependent on the thickness of the AZO electrode. A two times increase of the AZO electrode thickness results in the increase of current density *J*_sc_ from 3.21 to 9.79 mA/cm^2^ and open circuit voltage *U*_oc_ from 405 to 565 mV, while fill factor FF remains almost unchanged. Thus, PCE increases from 0.31% to 1.3%. It can be observed also, that characteristics of the devices fabricated on “Kapton” substrate, are independent on the AZO electrode thickness with a *J*_sc_ = 1.9 mA/cm^2^ and *U*_oc_ = 550 mV.

Only a few works have reported thin-film solar cells based on a-Si:H/PEDOT:PSS on a glass or flexible substrate. In Ref. [20] a hybrid p–i–n structure using pristine PEDOT:PSS on a glass substrate has been reported reaching a PCE = 2.1%. On the other hand, a flexible n–i–p type structure based on a-Si:H/PEDOT:PSS interface has been reported in Ref. [21] with a maximum efficiency of 6.52%. It is important to note that the structure reported in Ref. [21] was deposited on a non-transparent Ag-coated polyimide substrate and fabricated as substrate configuration. The advantage of use-transparent substrates, such as PEN and a pin-type structure, as reported in this work, is that the substrate can be simultaneously used as a supporting structure as well as a window for the capture of light (superstrate configuration). Some analysis of the junctions themselves fabricated on PEN and “Kapton” substrates will be given in the next section.

## 4. Discussion

**Figure 6:**
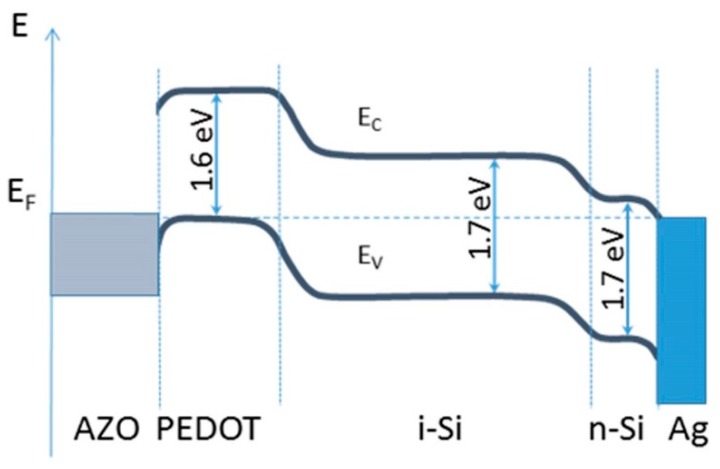
Electron energy diagram in thermal equilibrium for AZO/PEDOT:PSS/(i)Si:H/(n)Si:H/Ag structure.

Thermal equilibrium electron energy diagram in the studied hybrid structures is presented in Figure 6. The values of metals work functions, HOMO-LUMO energy positions of PEDOT:PSS and its work function ф were selected from data reported in literature [22,23]: LUMO PEDOT ф_LP_ = 3.6 eV; HOMO PEDOT ф_HP_ = 5.2 eV [22]; ф_Ag_ = 4.2 eV [23]; ф_AZO_ = 4.8 eV [24]. The values for optical gap *E*_g_, electron affinities χ and work functions ф for n-type and intrinsic a-Si layers were obtained in our previous experiments [25]: *E*_g_ = 1.7 eV; χ = 4.1 eV; ф_i-Si:H_ = 4.7 eV; ф_n-Si:H_ = 4.3 eV. It is worth noting, that the properties of interfaces (band bending and offsets) in such multilayer structure are of great importance and, hence, for the analysis of the photovoltaic performance the reliable data are needed. It would be better if these data could be obtained from measurements in device structures rather than measured in the films. To our knowledge, such data are not in literature and experimental techniques for them have not been developed yet. Therefore, we must use values available at present in literature to create some electron energy diagrams to provide at least some vision of the device performance.

It can be seen (Figure 6), that PEDOT layer acts as a p-type semiconductor with a large bandgap of ~1.6 eV. The band alignment in the studied structure favors the creation of an internal build-in electric field and prevents back diffusion of electrons into the frontal interface. Light absorption takes place mainly via band-to-band transitions in the intrinsic Si:H layer with the optical gap of *E*_g_ = 1.7 eV.

The difference in *J*_sc_ in the studied structures can arise from the following factors: (a) The difference in the amount of photons absorbed in the structure due to the different spectral transmission *T*(λ) of the substrates in the range of λ = 400–600 nm; (b) the difference in electrical properties of the films grown on different substrates and junctions between them. However, most probably the joint effect of both (a) and (b) factors contribute to the device performance.

**Figure 7:**
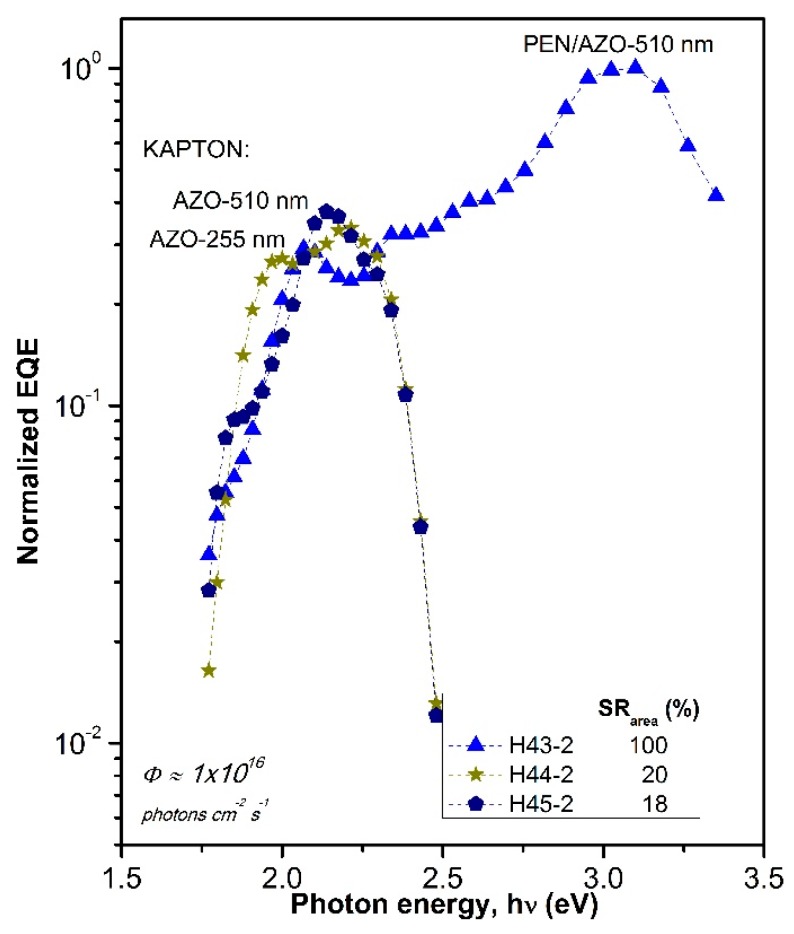
Spectral response of short circuit current density *J*_sc_ (h*ν*) for hybrid photovoltaic structures (1) H43-2; (2) 44-2; and (3) H45-2. Area under the curve (SR_area_) is indicated for the samples taking H43-2 SR_area_ as reference.

Since we are interested in finding out the contribution of frontal interface to the electrical properties of the junction, in order to distinguish between these two factors, spectral dependencies of short circuit current *J*_sc_ (λ) in fabricated flexible photovoltaic structures were measured and analyzed. Spectral characteristics *J*_sc_ (h*ν*) in the range of λ from 354 to 1200 nm for three hybrid structures (H43-2, H44-2, and H45-2) are presented in Figure 7.

In *J*_sc_ (h*ν*) dependence three regions can be distinguished: (1) low photon energy region (from hν = 1.8–2.2 eV) related to sub-gap absorption, determined mainly by properties of the intrinsic layer, (2) intermediate region (from h*ν* = 1.8 to 3.0 eV) where the current is a result of contributions from entire PV structure and (3) high photon energy region (h*ν* = 3.0 to 3.5 eV) where *J*_sc_ signal is mainly controlled by frontal interface properties. This last region and part of the region 2 are significantly shifted to low photon energy direction in the structure on “Kapton”, because of its spectral transmission (Figure 2). The loss of the short circuit current due to suboptimal light harvesting in the structure on “Kapton” substrate can be estimated, comparing the area under the corresponding curves of *J*_sc_ (λ) dependence (Figure 7): it is 20% in the structure H44-2 and 18% in H45-2, where 100% corresponds to the H43-2 device. However, it is interesting to note that short circuit current value *J*_sc_ (h*ν*) in region 1 and part of the region 2 is higher (nearly by the factor of 2) in the hybrid structure fabricated on “Kapton” substrate, even though the PEN substrate provides better optical transmission in the same range of wavelength. We tried to compare the junctions themselves fabricated on PEN and “Kapton” substrates by means of recalculation of performance J(U) characteristics for the structures on “Kapton” reducing them to those on PEN substrates. The factor for this recalculation is obtained from spectral characteristics of the real devices illuminated by AM 1.5 light. This analysis reveals differences in the junctions, mostly in the short circuit current density (about 15% less for the junction on “Kapton” substrate) while open circuit voltage shows practically the same value. We believe that worse junction properties of “Kapton”-based device could arise from the morphological features of AZO surface deposited on “Kapton”, characterized by higher roughness and by the presence of sharp peaks of AZO, as shown by our AFM data. Such peaks can produce additional stress on the film grown on top of it and in this way contribute to the deterioration of electronic properties of the junction. In addition, these peaks could be responsible for the creation of parasitic shunts across the junction deteriorating device performance.

## 5. Conclusions

We have fabricated and studied hybrid organic/inorganic p–i–n PV devices on flexible plastic substrates. The device structures consist of the frontal electrode of transparent conductive oxide (Al-doped zinc oxide, AZO); organic p-type semiconductor (PEDOT:PSS); intrinsic and n-doped amorphous Si:H layer and the silver rear electrode. These materials have been deposited by different techniques: AZO and Ag electrodes by sputtering, organic semiconductor by spin- coating, and intrinsic and n-doped Si:H by PECVD. “Kapton” and PEN foils were used as the substrates. Fabrication temperature did not exceed *T* < 165 °C. All the materials, techniques and regimes used for fabrication proved to be compatible with each other allowing fabrication of multilayered device structures. The fabrication of the device structures started with AZO deposition; therefore, its surface morphology has been studied by AFM. The AFM results showed that surface morphological characteristics of AZO are affected by the substrates type. SEM characterization has demonstrated the complexity of the material and interface of the structures, as well as an effect of “healing” of AZO surface by the organic semiconductor. PV hybrid structure on PEN substrate showed the highest value of short circuit current density *J*_sc_ = 9.79 mA/cm^2^, *U*_oc_ = 565 mV and PCE η = 1.3%. This superior performance compared to that of the devices on “Kapton” substrate is mainly due to the wider optical transmission window of the PEN substrate. However, device diagnostics using the spectral dependence of the short circuit current has revealed that p–i–n junction of “Kapton”-based device itself provides about 15% less current that that of a PEN-based device. We believe that the inferior junction properties of “Kapton”-based device could arise from the morphological features of AZO surface deposited on “Kapton”, characterized by higher roughness and by the presence of sharp peaks of AZO.

## Figures and Tables

**Table 1:** Morphological characteristics of PEN and “Kapton” substrates and AZO films of 255 and 510 nm thickness deposited on top of the substrates.

Substrate	PEN			“Kapton”			Glass
Process ID	Substrate	H42	H43	Substrate	H44	H45	REF
	Deposited Films						
	No Film Deposited	AZO 255 nm	AZO 510 nm	No Film Deposited	AZO 255 nm	AZO 510 nm	AZO 510 nm
RMS Roughness *R*_q_ (nm)	1.20 + 0.06	1.39 + 0. 07	1.00 + 0.06	1.07 + 0.05	1.85 + 0.1	1.41 + 0.7	1.19 + 0.06
Surface Skewness *S*_k_	0.11 + 0.05	0.15 + 0.006	−0.21 + 0.01	−0.26 + 0.01	0.37 + 0.02	3.02 + 0.15	0.05 + 0.002
Surface Kurtosis *K*_u_	2.84 + 0.14	3.15 + 0.15	2.01 + 0.02	3.42 + 0.2	4.8 + 0.24	7.63 + 0.40	3.39 + 0.17

**Table 2:** Summary of the performance characteristics of the photovoltaic devices.

Sample	Frontal Interface	*J*_sc_ (mA/cm^2^)	*U*_oc_ (V)	FF	PCE (%)
H41-1	Reference	10.04	0.565	0.26	1.47
H42-2	PEN/AZO-255 nm	3.21	0.405	0.239	0.31
H43-2	PEN/AZO-510 nm	9.79	0.565	0.236	1.3
H44-2	KAPTON/AZO-255 nm	1.95	0.55	0.234	0.25
H45-2	KAPTON/AZO-510 nm	1.9	0.55	0.23	0.24
